# Paradigm Shifting Research: Key Studies in Urologic Oncology

**DOI:** 10.1245/s10434-023-14838-w

**Published:** 2024-02-01

**Authors:** Shawn Dason, Cheryl T. Lee

**Affiliations:** Department of Urology, The Ohio State University, Columbus, OH USA

## Abstract

**Background:**

Genitourinary malignancies have a substantial impact on men and women in the USA as they include three of the ten most common cancers (prostate, renal, and bladder). Other urinary tract cancers are less common (testis and penile) but still have profound treatment implications related to potential deficits in sexual, urinary, and reproductive function. Evidenced-based practice remains the cornerstone of treatment for urologic malignancies.

**Methods:**

The authors reviewed the literature in consideration of the four top articles influencing clinical practice in the prior calendar year, 2022.

**Results:**

The PROTECT trial demonstrates favorable 15-years outcomes for active monitoring of localized prostate cancer. The SEMS trial establishes retroperitoneal lymph node dissection as a viable option for patients with seminoma of the testis with limited retroperitoneal lymph node metastases. CheckMate 274 supports adjuvant immunotherapy following radical cystectomy for muscle-invasive bladder cancer with a high risk of recurrence. Data reported from the IROCK consortium reinforce stereotactic ablative radiotherapy as an option for localized renal cell carcinoma.

**Conclusion:**

The care for patients with urologic cancers has been greatly improved through advances in surgical, medical, and radiation oncologic treatments realized through prospective randomized clinical trials and large multicenter collaborative groups.

Urologic cancers include three of the ten most common malignancies in the USA.^1^ Surgical, medical, and radiation oncology advances in the past year have advanced the care of patients with genitourinary cancers. In this review, we discuss the four articles that most informed our practice in the past year and highlight key studies that have challenged historical treatment norms across four genitourinary malignancies.

## PROTECT

The PROstate TEsting for Cancer and Treatment trial reports 15-year outcomes from a randomized clinical trial examining treatment for localized prostate cancer.^2^ Prostate cancer is the most common non-skin malignancy in American men, with 288,300 new cases estimated in 2023.^1^ Although highly prevalent, many cases of prostate cancer are indolent and unlikely to result in mortality due to other competing causes of mortality in older men. Screening tools for prostate cancer, such as serum prostate specific antigen (PSA), greatly increased early detection of the disease, but a real recognition of prostate cancer overtreatment also emerged. Overtreatment of indolent or low risk prostate cancer can result in urinary, sexual, and bowel toxicity—all from a cancer unlikely to threaten survival. Nonetheless, prostate cancer remains the second most common cause of cancer death in American men^1^ and treating appropriate populations is known to improve survival.^3–5^ Striking the correct balance between overtreatment and appropriate treatment has been a large focus of prostate cancer care in recent decades.

The PROTECT study^2^ was a clinical trial conducted in the United Kingdom enrolling participants between 1999 and 2009. A total of 1643 men with prostate cancer were randomized to receive surgery, radiation therapy, or active monitoring. Those initially assigned to active monitoring could cross over to treatment groups on the basis of disease progression.

The study population of PROTECT was a predominantly low-risk prostate cancer population; 77.2% of participants had a Gleason score of 6 and 76% had a stage of T1c (PSA detected nonpalpable cancers). Nonetheless, 24.1% of participants had intermediate-risk disease and 9.6% had high-risk prostate cancer.

At a median follow-up of 15 years, death from prostate cancer occurred in 45 men (2.7%). Prostate cancer survival at 15 years in the active monitoring, surgery, and radiation groups was 96.6%, 97.2%, and 97.7%, respectively; no statistical difference in disease-specific survival was observed between groups (Table 1). Death from any cause occurred in 21.7% of the population with a similar distribution across the groups.

**Table 1:** Primary and secondary outcome measures of the PROTECT study, the active monitoring crossover rate to radiotherapy or prostatectomy was 61.1% at 15 years

Outcome and trial group	No of events	No. of person-Yr	Rate per 1000 person-Yr (95% CI)	Hazard ratio (95% CI)*
Primary outcome				
Death from prostate cancer†				
Active monitoring	17	7633	2.2 (1.4–3.6)	Reference
Prostatectomy	15	7766	1.5 (0.9–2.7)	0.66 (0.31–1.39)
Radiotherapy	16	7628	2.1 (1.3–3.4)	0.88 (0.44–1.74)
Secondary outcomes				
Death from any cause				
Active monitoring	124	7633	16.2 (13.6–19.3)	Reference
Prostatectomy	117	7766	15.0 (12.5–18.0)	0.89 (0.69–1.15)
Radiotherapy	115	7628	15.0 (12.5–18.0)	0.88 (0.68–1.13)
Metastatic disease				
Active monitoring	51	7324	7.1 (5.4–9.3)	Reference
Prostatectomy	26	7594	3.5 (2.4–5.1)	0.47 (0.29–0.76)
Radiotherapy	27	7467	3.7 (2.5–5.4)	0.48 (0.30–0.77)
Androgen-deprivation therapy				
Active monitoring	69	7197	9.4 (7.4–11.9)	Reference
Prostatectomy	40	7452	5.3 (3.9–7.2)	0.54 (0.37–0.80)
Radiotherapy	42	7328	5.6 (4.2–7.6)	0.54 (0.36–0.79)
Clinical progression‡				
Active monitoring	141	6596	21.4 (18.1–25.2)	Reference
Prostatectomy	58	7258	8.0 (6.2–10.3)	0.36 (0.27–0.49)
Radiotherapy	60	7173	8.4 (6.5–10.8)	0.5 (0.26–0.48)

Fifteen-Year Outcomes after Monitoring, Surgery, or Radiotherapy for Prostate Cancer. Reprinted with permission.

^*^Hazard ratio are estimated after adjustment for trail center, patient’s age at baseline, Gleason score, and prostate specific antigen level at baseline (log-transformed). The widths of confidence intervals for secondary outcomes have not been adjusted for multiplicity and cannot be used in place of hypothesis is testing.

^†^The primary outcomes are definite or probable cancer morality, as adjudicated by an independent cause of death committee. *P*  =  0.53 for the primary-outcome comparison

^‡^Disease progression included evidence of metastatic disease, the initiation of long-term androgen-deprivation therapy, diagnosis of clinical T3 or T4 disease, ureteric obstruction, rectal fistula, or urinary catheterization because of tumor growth

Metastasis-free survival in the active monitoring, surgery, and radiation groups was 90.6%, 95.3%, and 95%, respectively. This indicates that, although uncommon, development of metastatic prostate cancer was approximately twice as likely in men initially managed with active monitoring. By 15 years, 61.1% of those initially undergoing active monitoring had received radical treatment.

In a paired publication, 12-years quality of life outcomes for this study were reported.^6^ Overall quality of life was similar between groups. Those receiving surgery were more likely to have urinary incontinence requiring pads (18–24%) than the active monitoring (9–11%) or radiotherapy (3–8%) groups. Erectile function did not differ between study groups. Fecal leakage affected 12% in the radiotherapy group compared with 6% in the other groups.

The clinical implications of PROTECT are broad:

1. PROTECT supports active surveillance as the preferred management strategy for men with low-risk prostate cancer.^7^ Modern active surveillance protocols differ slightly from the active monitoring approach utilized in PROTECT. Some advances in active surveillance that have arisen since the trial era include more routine use of magnetic resonance imaging (MRI) and genomic classifiers, reclassification of cribriform pattern histology to Gleason 7, increasing recognition of germline risk factors, improved definitions of progression, and better treatment options at the time of progression. Nonetheless, despite being less developed than modern active surveillance, mortality was not higher with active monitoring in PROTECT compared with upfront treatment. Although there was a higher rate of metastatic disease noted with active monitoring, it is likely that the more vigilant approach of active surveillance would limit this risk. Modern active surveillance series demonstrate very low (1–2%) long term rates of metastatic disease.^8^
2. PROTECT provides rationale for prostate cancer treatment in those with clinically significant disease and a long life expectancy. Although a survival benefit was not seen in the favorable population of PROTECT, there was still a lower rate of metastatic disease, androgen deprivation therapy use, and clinical progression in patients receiving upfront treatment. Median survival of metastatic prostate cancer can exceed 5 years,^9^ but it is still eventually fatal and impacts quality of life. The 61.1% crossover from active monitoring to surgery or radiation during the study period occurred when unfavorable disease was noted, making crossover critical in this group achieving a similar survival to the radiotherapy and prostatectomy at 15 years. The importance of crossover to study outcomes is suggested by prior studies in less favorable localized prostate cancer populations that have demonstrated improved survival with local treatment.^3–5^ It is of utmost importance that PROTECT is not misconstrued as endorsing therapeutic nihilism toward all patients with prostate cancer. Prostate cancer remains the second most common cause of cancer death in men in the USA.^1^ A thoughtful approach is essential when considering the specific patient and disease factors that will permit active surveillance or prompt treatment.
3. PROTECT suggests that prostatectomy and radiation therapy offer similar oncologic outcomes but differ in their individual side effect profiles. This is the first randomized trial to support this conventional wisdom in prostate cancer management. Radiation and prostatectomy resulted in similar improvements in secondary endpoints such as metastasis-free survival, receipt of androgen deprivation therapy, and freedom from progression. While overall quality of life was similar, the higher rates of urinary incontinence with prostatectomy are contrasted to the higher rates of bowel toxicity with radiation. Unsurprisingly, most patients did not retain erectile function in the long term. While these findings cannot be extrapolated to specific populations (e.g., very-high-risk prostate cancer or oligometastatic prostate cancer), they likely apply to most patients with localized prostate cancer.
4. PROTECT supports the adage that many men have heard upon their diagnosis of low- and intermediate-risk prostate cancer—“you’re more likely to die *with* prostate cancer than to die *from* prostate cancer.” PROTECT demonstrated that men with prostate cancer were eight times more likely to die from something other than prostate cancer. Additionally, metastatic prostate cancer developed in < 10% of patients in this study. This study affirms a modern-day practice of active surveillance in the overwhelming proportion of men with low-risk disease and a substantial number of men with intermediate-risk disease.

## SEMS

Surgery in Early Metastatic Seminoma is a phase II study that establishes retroperitoneal lymph node dissection as a central treatment in patients with testicular seminoma with limited retroperitoneal lymph node metastases, expanding traditional therapeutic options beyond systemic chemotherapy or radiation.^10^ Testicular cancer is the most common solid organ malignancy in men aged 20–40 years, with an estimated 9190 new diagnoses in the USA in 2023.^1^ Survival rates for testicular cancer have markedly improved in the past 50 years and exceed 95%.^11^ Although necessary to achieve this high cure rate, chemotherapy and radiation therapy regimens are associated with a higher rate of cardiovascular disease and secondary malignancies decades after treatment.^12^ These survivorship concerns are important since testicular cancer survivors comprise the eighth largest group of male cancer survivors given their young age at diagnosis and high survival rate.^13^

Most testicular cancers are germ cell tumors, which can be divided into seminoma and non-seminoma. Non-seminomatous germ cell tumors (NSGCT) with limited retroperitoneal lymph node metastases can be offered retroperitoneal lymph node dissection (RPLND) or chemotherapy, both with a high primary cure rate. The appeal of primary RPLND in this setting is the avoidance of long-term toxicities with the exception of retrograde ejaculation in some cases. However, seminomas with limited retroperitoneal metastases have historically been managed with chemotherapy or radiation, both of which have the potential for long-term toxicity, including infertility, low testosterone, pulmonary compromise, hypertension, coronary artery disease, metabolic syndrome, and secondary malignancy. Increasing recognition of long-term toxicities in patients receiving chemotherapy and/or radiation has prompted a number of groups to investigate RPLND for patients with limited seminomatous retroperitoneal metastases.

The SEMS trial was a prospective multi-institutional study that enrolled 55 men with a diagnosis of seminoma with up to two enlarged retroperitoneal lymph nodes with a maximal dimension of 3 cm or less. Participants underwent RPLND with a template at the discretion of the operating surgeon. RPLNDs were performed in centers of excellence by credentialed surgeons.

**Fig. 1:**
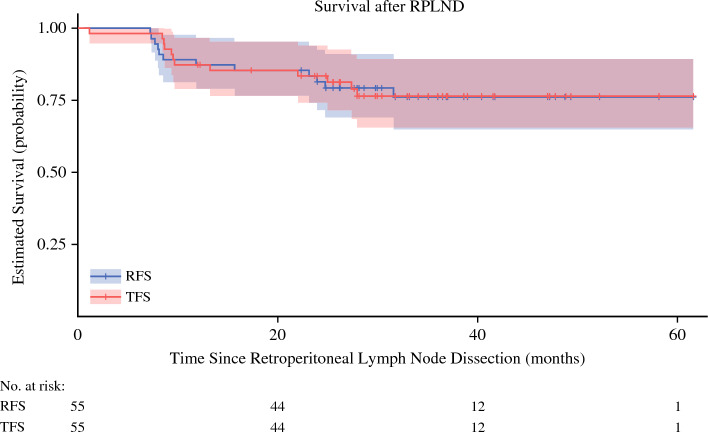
Recurrence-free survival following primary retroperitoneal lymph node dissection in early metastatic seminoma. *Note* Reprinted with permission

After a median follow-up of 33 months after surgery, recurrence-free survival was 81% (Fig. 1). Of those that recurred, all participants were salvaged with chemotherapy (10/12) or repeat RPLND (2/12). All 55 participants were free of disease at last follow-up.

Perioperative complications were limited—only one participant sustained a Clavien–Dindo grade 3 or higher complication (chylous ascites leak) in the early perioperative period. Other long-term complications from RPLND were noted in four participants [asymptomatic incisional hernia (*n* = 1) and anejaculation (*n* = 3)].

The SEMS trial demonstrates a similar recurrence-free survival for RPLND in seminoma with limited retroperitoneal lymph node metastasis as two other prospective studies published this year (COTRIMS and PRIMETEST).^14,15^ These data taken together provide strong rationale to consider RPLND as part of shared decision-making for this clinical state. RPLND is appealing over alternative options because of its limited long-term risks beyond retrograde ejaculation. The limitations of RPLND are its lower recurrence-free survival as compared with historical observations in patients treated with chemotherapy or radiation. Another important consideration for RPLND relates to its technical complexity, essentially requiring access to a high-volume surgeon and a high-volume center to realize ideal outcomes. This could represent a significant barrier, particularly for patients in rural communities.

The technique for performing RPLND in seminoma differs by surgeon:

1. While SEMS did not find an association between RPLND template (bilateral versus unilateral) and disease recurrence, prior data in patients with non-seminomatous disease has raised the concern of increased risk of retroperitoneal relapse with unilateral template dissections.^16^ Because all studies for RPLND in seminoma have predominantly used a unilateral template, it is unclear whether routinely performing a bilateral template RPLND would raise recurrence-free survival rates following RPLND to the 90%+ expected from non-surgical approaches. Notably, 5/12 recurrences in SEMS were in the retroperitoneum—these might have been avoided if a bilateral template and/or more thorough dissection were performed at the time of the original RPLND.
2. Robotic RPLND is associated with a more rapid recovery than the conventional open approach. The ability to return to regular life faster following treatment is particularly appealing to this young population. Modern robotic RPLND, when performed by a urologic oncologist who also performs open RPLND in complex post-chemotherapy cases is identical to the open approach except for the pneumoperitoneum. Widespread robotic RPLND adoption has been hampered by case reports of aberrant recurrence patterns^17^ and it was not used in SEMS. It will be important to understand whether aberrant patterns of recurrence occur with robotic RPLND for seminoma when performed by those with sufficient expertise. While aberrant recurrences are a serious concern, salvage may be slightly easier with seminoma given its chemo- and radiotherapy responsiveness relative to NSGCT histologies such as teratoma.

## CheckMate 274

CheckMate 274 is a multicenter phase III randomized clinical trial that demonstrated a survival advantage for adjuvant nivolumab immunotherapy, as compared with placebo, following radical surgery for muscle invasive urothelial carcinoma of the bladder or upper tract.^18^ Bladder cancer is the sixth most common cancer in the USA, with an estimated 82,290 cases in 2023.^1^ Patients with non-metastatic muscle-invasive bladder cancer (MIBC) are conventionally managed with radical cystectomy. Neoadjuvant platinum-based chemotherapy is known to provide a 5% survival benefit following radical cystectomy.^19^ Unfortunately many patients with MIBC are not candidates for neoadjuvant chemotherapy; neoadjuvant chemotherapy is received by less than 60% of patients even in centers of excellence.^20^ Moreover, adjuvant chemotherapy has not conclusively demonstrated a survival benefit^21^ in patients with MIBC undergoing radical cystectomy, and prolonged recovery from radical cystectomy may render a patient less able to receive adjuvant chemotherapy.

Immune checkpoint inhibition with monoclonal antibodies against PD-1 or PD-L1 is known to have activity in metastatic bladder cancer.^22^ Immune checkpoint inhibitors are particularly appealing in this patient population because these agents do not result in nephrotoxicity and have a more favorable toxicity profile than platinum-based chemotherapy.

**Fig. 2:**
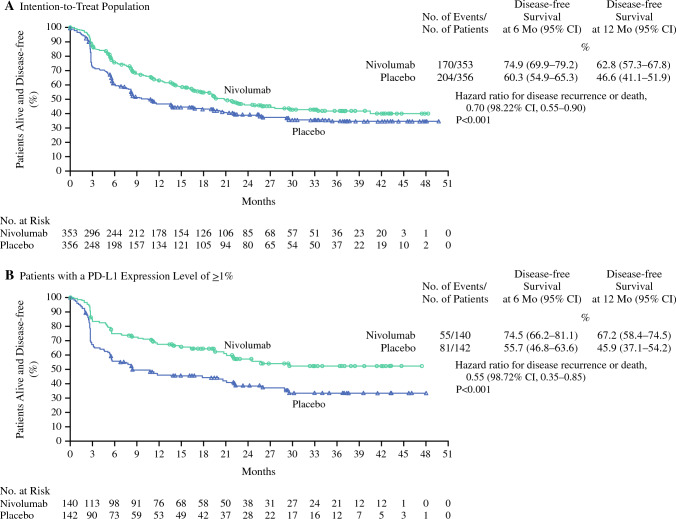
Primary outcomes of Checkmate 274. *Source* Adjuvant Nivolumab versus Placebo in Muscle-Invasive Urothelial Carcinoma. *Note* Reprinted with permission

Bajorin et al. recently reported CheckMate 274, a randomized clinical trial that explored the role of adjuvant nivolumab (anti-PD1) for 1 year after radical surgery for high-risk urothelial carcinoma; nearly 80% underwent cystectomy.^18^ In this study, 709 participants at high risk of recurrence after radical cystectomy or nephroureterectomy were randomly allocated to receive adjuvant nivolumab or placebo. The primary endpoint was recurrence-free survival. Those that received adjuvant nivolumab had a recurrence-free survival of 74.9% compared with 60.3% for those receiving placebo (HR 0.7, 95% CI 0.55–0.90). Cancer-specific survival outcomes have not yet been reported (Fig. 2).

Toxicity in the nivolumab group included three treatment-related deaths due to pneumonitis (*n* = 2) and bowel perforation (*n* = 1). Grade 3 toxicities in the nivolumab group were observed in 17.9% of subjects; all grade toxicities in the nivolumab arm were noted in 77.5%. The most common treatment-related toxicities were pruritis, fatigue, diarrhea, and rash.

CheckMate 274 is significant because it supports a paradigm shift in the treatment of high-risk patients following radical surgery. Traditionally these patients may have received adjuvant chemotherapy but may also have been managed expectantly. These data do not obviate the need for neoadjuvant chemotherapy, as the evidence supporting that approach remains robust.^19^ Nonetheless, for patients with persistent MIBC and/or node-positive disease after neoadjuvant chemotherapy or those chemo-naïve patients with extravesical disease and ineligible for or declining neoadjuvant chemotherapy, adjuvant nivolumab clearly reduces recurrence risk. Given the conflicting data and difficulty in administering adjuvant chemotherapy, adjuvant nivolumab has become standard treatment for these high-risk patients following radical cystectomy.

Application of these data is limited by the early nature of the study outcomes. It is unclear if positive recurrence-free survival outcomes in CheckMate 274 will translate into improved cancer-specific survival. While response rates to immune checkpoint inhibition in urothelial carcinoma is low, patients that achieve a response can have prolonged survival. Still, the same participants that benefited from adjuvant nivolumab in CheckMate 274 might have similarly benefited from early salvage treatment with an immune checkpoint inhibitor. Tolerance of toxicity and travel to a medical facility for an infusion every 2 weeks can also be difficult in this patient population.

Interestingly, CheckMate 274 included patients with upper tract urothelial carcinoma (i.e., urothelial carcinoma of the renal pelvis or ureter). Patients with cancer of the renal pelvis or ureter have a similar histology to bladder cancer (urothelial carcinoma) and are usually managed with radical nephroureterectomy. Adjuvant chemotherapy following radical nephroureterectomy is supported by the POUT study, which demonstrated a recurrence-free survival benefit of platinum-based adjuvant chemotherapy over placebo.^23^ The main concern with adjuvant chemotherapy following radical nephroureterectomy is that many patients are rendered platinum-ineligible following surgery due to declines in renal function and exposure to a nephrotoxic agent. Consequently, a non-nephrotoxic adjuvant option would be attractive in high-risk patients treated with radical nephroureterectomy.

In CheckMate 274, nivolumab did not improve recurrence-free survival in the subgroups with ureteral (HR 1.56, 95% CI 0.70–3.48) or renal pelvic (HR 1.23, 95% CI 0.67–2.23) cancers. Notably, only 21% of the participants in CheckMate 274 had upper tract urothelial carcinoma, rendering these subgroups underpowered. These findings indicate that adjuvant platinum-based chemotherapy remains the best option for adjuvant treatment following radical nephroureterectomy. However, based on the overall CheckMate 274 population, nivolumab is a reasonable adjuvant option for patients who are not eligible for platinum-based chemotherapy following radical nephroureterectomy.

## International Radiosurgery Oncology Consortium for Kidney (IROCK) Collaborative

Stereotactic ablative radiotherapy in localized renal cell carcinoma is supported by promising intermediate-term data from the International Radiosurgery Oncology Consortium for Kidney (IROCK) Collaborative multicenter, multinational series of prospective and retrospective data.^24^ Kidney cancer is the seventh most common cancer in the USA with an estimated 81,800 cases in 2023.^1^ Most localized kidney cancers are histologically renal cell carcinoma (RCC), have a favorable prognosis, and are managed with surgical resection (i.e., partial or radical nephrectomy). In the healthy patient, surgical management has low complication rates.

One-third of RCCs are diagnosed in patients over the age of 75 years,^25^ suggesting that many patients diagnosed with RCC will have an elevated surgical risk and/or chronic kidney disease. Competing causes of mortality also overshadow RCC-related mortality in the older or comorbid patient. Fortunately, active surveillance can usually be safely employed for small RCCs in an older or comorbid patient as an initial management strategy. We continue to search for a good option for RCC management in the patient who is not a good candidate for either surgical management or active surveillance.

Stereotactic ablative radiotherapy (SABR) has challenged the conventional wisdom that RCC is a radioresistant cancer. This philosophy was based on the limited efficacy of standard radiotherapy fractionation in the treatment of RCC. However, SABR involves treatment with a high dose per fraction and a limited number of fractions (typically five or less). The appeal of SABR is its completely non-invasive approach, which can be applied, selectively, to patients with significant medical comorbidities who are suboptimal candidates for surgery or active surveillance.

These common clinical scenarios encountered by the urologic oncologist include:

1. A high-risk surgical candidate with a mass felt to have an elevated risk for metastatic disease in the patient’s expected lifespan (e.g., due to size, growth from prior images, aggressive histology on biopsy, or appearance of local invasion or venous tumor thrombus), or
2. A high-risk surgical candidate with a mass progressing on active surveillance.

**Fig. 3:**
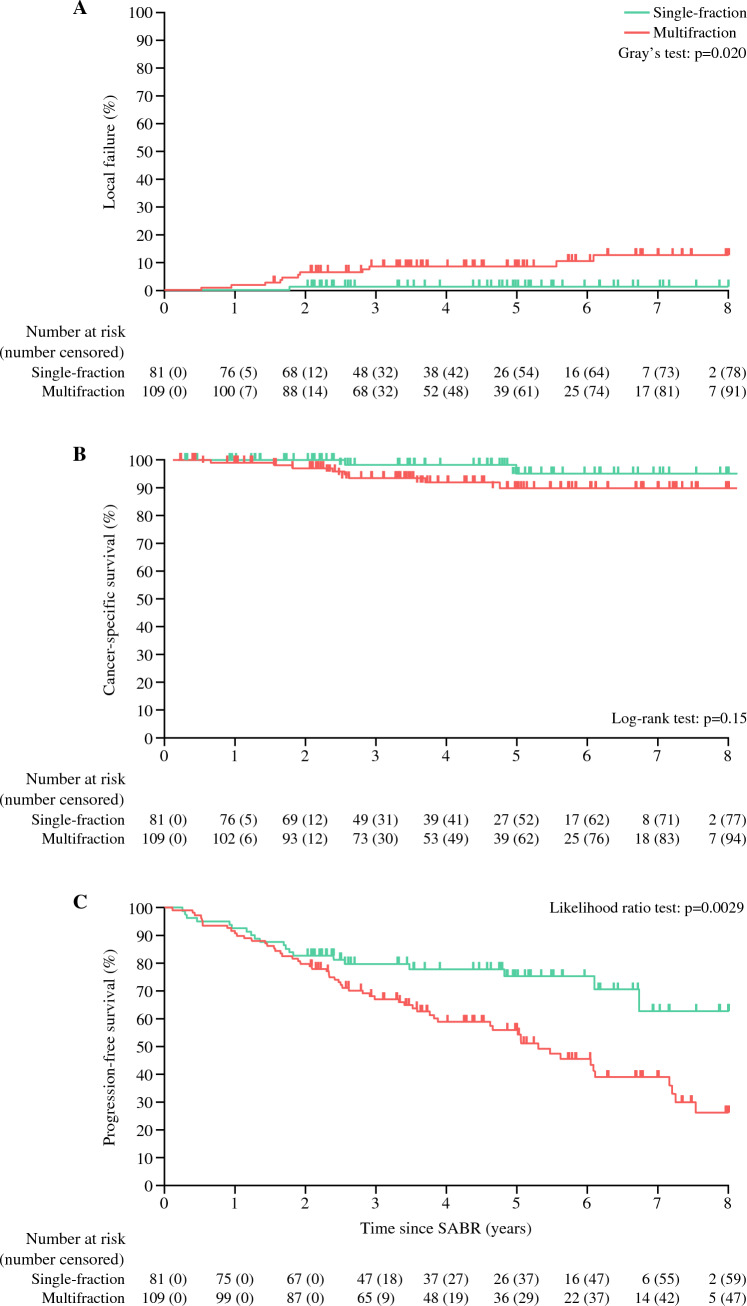
Oncologic outcomes following stereotactic ablative radiotherapy for renal cell carcinoma. *Note*: Reprinted with permission

Efficacy of SABR has been supported by the recent publication of 5-year outcomes from the IROCK Collaborative. In this multi-institutional report, 190 patients with RCC were treated with SABR. Patients had a median follow-up of 5.0 years. In total, 83% of patients received a biopsy, which revealed clear cell RCC in 85% of this subgroup. The median tumor diameter treated was 4.0 cm. Local failure rate at 5 years was 5.5%. Cancer-specific survival at 5 years was 92.0% (Fig. 3). In those with tumor recurrence, the pattern of disease was local in 2%, distant in 7%, and both local and distant in 4%. Of the local-only failures, one was amenable to radical nephrectomy and three were inoperable due to medical comorbidities or T4 disease.

Treatment-related toxicities were limited with SABR. The only high-grade toxicity was a single grade 4 gastritis. Grade 1–2 toxicities were noted in 37%, the most common of which were fatigue (27%), nausea (13%), and chest wall pain (6%). Renal functional decline was 14.2 ml/min GFR at 5 years, which is hard to separate from natural decline in this population with a median GFR prior to SABR of 60 ml/min.

This IROCK series presents early evidence for an ideological shift in the treatment of localized RCC. Although retrospective in nature, the data are provocative and will surely generate prospective studies, particularly given the exceedingly low cancer-specific mortality and the high rates of local control. On the basis of these data, urologic oncologists will be driven to enter patients into prospective clinical trials to confirm the long-term benefits for SABR as a viable treatment strategy for localized RCC.

The biggest uncertainty surrounding SABR in localized RCC is when it should be used. The ease and non-invasive nature of SABR could result in overuse. Surveillance is still preferable to SABR in the comorbid patient with a small renal mass, as the metastatic rate with surveillance of a small renal mass is 1% in the short term.^26^

Assessment of SABR efficacy is also difficult given the favorable natural history of RCC. This study lacked a control group—and it is likely that most of the favorable tumors treated in this study would not have metastasized in a 5-year timespan if left untreated. Local control was assessed according to RECIST criteria and tumor biopsies are generally not performed following SABR for RCC. Given the lack of a good alternative in the populations that SABR is being used in, these limitations do not diminish the impact of the study. Nonetheless, it is unclear whether local progression-free survival by RECIST criteria truly predicts metastasis-free or cancer-specific survival in the long term. Moreover, in the surgical candidate, it would be a dangerous extrapolation of this study to offer SABR as an alternative to surgery for the unfavorable masses we commonly see in practice.

This study is also seminal in expanding the role of stereotactic ablative radiotherapy in renal cell carcinoma beyond localized RCC. SABR is being studied across the disease spectrum of RCC, including:

1. Primary or neoadjuvant treatment for patients with inferior vena cava tumor thrombus^27–29^
2. Metastasis directed therapy as an alternative to metastasectomy^30^
3. Cytoreductive treatment as an alternative to cytoreductive nephrectomy^31^
4. Treatment of oligoprogressive disease to prolong a response to systemic therapy^32^

## Conclusion

The past year has seen practice-transforming research that has enhanced the standards of care for patients with urologic cancer.

## References

[1] Siegel RL, Miller KD, Wagle NS, Jemal A (2023). Cancer statistics, 2023. CA Cancer J Clin.

[2] Hamdy FC, Donovan JL, Lane JA (2023). Fifteen-year outcomes after monitoring, surgery, or radiotherapy for prostate cancer. N Engl J Med.

[3] Bill-Axelson A, Holmberg L, Garmo H (2018). Radical prostatectomy or watchful waiting in prostate cancer: 29-Year Follow-up. N Engl J Med.

[4] Widmark A, Klepp O, Solberg A (2009). Endocrine treatment, with or without radiotherapy, in locally advanced prostate cancer (SPCG-7/SFUO-3): an open randomised phase III trial. Lancet.

[5] Warde P, Mason M, Ding K (2011). Combined androgen deprivation therapy and radiation therapy for locally advanced prostate cancer: a randomised, phase 3 trial. Lancet.

[6] Donovan JL, Hamdy FC, Lane JA (2023). Patient-reported outcomes 12 years after localized prostate cancer treatment. NEJM Evid.

[7] Guidelines Detail. NCCN. Accessed November 15, 2022. https://www.nccn.org/guidelines/guidelines-detail

[8] Sanda MG, Cadeddu JA, Kirkby E (2018). Clinically localized prostate cancer: AUA/ASTRO/SUO Guideline: Part I: risk stratification, shared decision making, and care options. J Urol.

[9] James ND, Clarke NW, Cook A (2022). Abiraterone acetate plus prednisolone for metastatic patients starting hormone therapy: 5-year follow-up results from the STAMPEDE randomised trial (NCT00268476). Int J Cancer.

[10] Daneshmand S, Cary C, Masterson T (2023). Surgery in early metastatic seminoma: a phase II trial of retroperitoneal lymph node dissection for testicular seminoma with limited retroperitoneal lymphadenopathy. JCO.

[11] Hanna N, Einhorn LH (2014). Testicular cancer: a reflection on 50 years of discovery. JCO..

[12] Fung C, Dinh PC, Fossa SD, Travis LB (2019). Testicular cancer survivorship. J Nat Compr Cancer Netw.

[13] Miller KD, Nogueira L, Devasia T (2022). Cancer treatment and survivorship statistics, 2022. CA Cancer J Clin.

[14] Hiester A, Che Y, Lusch A (2023). Phase 2 single-arm trial of primary retroperitoneal lymph node dissection in patients with seminomatous testicular germ cell tumors with clinical stage IIA/B (PRIMETEST). Eur Urol..

[15] Heidenreich A, Paffenholz P, Hartmann F, Seelemeyer F, Pfister D (2023). Retroperitoneal lymph node dissection in clinical stage IIA/B metastatic seminoma: results of the COlogne Trial of Retroperitoneal Lymphadenectomy In Metastatic Seminoma (COTRIMS). Eur Urol Oncol.

[16] Eggener SE, Carver BS, Sharp DS, Motzer RJ, Bosl GJ, Sheinfeld J (2007). Incidence of disease outside modified retroperitoneal lymph node dissection templates in clinical stage I or IIA nonseminomatous germ cell testicular cancer. J Urol.

[17] Calaway AC, Einhorn LH, Masterson TA, Foster RS, Cary C (2019). Adverse surgical outcomes associated with robotic retroperitoneal lymph node dissection among patients with testicular cancer. Eur Urol.

[18] Bajorin DF, Witjes JA, Gschwend JE (2021). Adjuvant nivolumab versus placebo in muscle-invasive urothelial carcinoma. N Engl J Med.

[19] Vale CL (2005). Neoadjuvant chemotherapy in invasive bladder cancer: update of a systematic review and meta-analysis of individual patient data: advanced bladder cancer (ABC) meta-analysis collaboration. Eur Urol.

[20] Almassi N, Cha EK, Vertosick EA (2020). Trends in management and outcomes among patients with urothelial carcinoma undergoing radical cystectomy from 1995 to 2015: the Memorial Sloan Kettering experience. J Urol.

[21] Vale CL (2005). Adjuvant chemotherapy in invasive bladder cancer: a systematic review and meta-analysis of individual patient data: advanced bladder cancer (ABC) meta-analysis collaboration. Eur Urol.

[22] Bellmunt J, de Wit R, Vaughn DJ (2017). Pembrolizumab as second-line therapy for advanced urothelial carcinoma. N Engl J Med.

[23] Birtle A, Johnson M, Chester J (2020). Adjuvant chemotherapy in upper tract urothelial carcinoma (the POUT trial): a phase 3, open-label, randomised controlled trial. Lancet.

[24] Siva S, Ali M, Correa RJM (2022). 5-year outcomes after stereotactic ablative body radiotherapy for primary renal cell carcinoma: an individual patient data meta-analysis from IROCK (the International Radiosurgery Consortium of the Kidney). Lancet Oncol.

[25] Capitanio U, Bensalah K, Bex A (2019). Epidemiology of renal cell carcinoma. Eur Urol.

[26] Jewett MAS, Mattar K, Basiuk J (2011). Active surveillance of small renal masses: progression patterns of early stage kidney cancer. Eur Urol.

[27] Freifeld Y, Pedrosa I, Mclaughlin M (2022). Stereotactic ablative radiation therapy for renal cell carcinoma with inferior vena cava tumor thrombus. Urol Oncol Semin Orig Invest.

[28] Freifeld Y, Margulis V, Woldu SL, Timmerman R, Brugarolas J, Hannan R (2019). Stereotactic body radiation therapy for renal cell carcinoma with inferior vena cava thrombus: initial experience report and literature review. Kidney Cancer.

[29] Dason S, Mohebali J, Blute ML, Salari K (2023). Surgical management of renal cell carcinoma with inferior vena cava tumor thrombus. Urol Clin North Am.

[30] Dason S, Lacuna K, Hannan R, Singer EA, Runcie K (2023). State of the art: multidisciplinary management of oligometastatic renal cell carcinoma. Am Soc Clin Oncol Educ Book.

[31] Ray S, Dason S, Singer EA (2023). Integrating surgery in the multidisciplinary care of advanced renal cell carcinoma. Urol Clin North Am.

[32] Schoenhals JE, Mohamad O, Christie A (2021). Stereotactic ablative radiation therapy for oligoprogressive renal cell carcinoma. Adv Radiat Oncol.

